# COVID-19-Related Risk Factors for Developing Occupational Contact Dermatitis Along With Its Incidence, Prevention, and Management: A Systematic Review

**DOI:** 10.7759/cureus.68441

**Published:** 2024-09-02

**Authors:** Esraa M AlEdani, Jahnavi Gurramkonda, Shaan Chaudhri, Amina Amin, Binay K Panjiyar, Dhuha S Al-taie, Tuheen Sankar Nath

**Affiliations:** 1 Dermatology, California Institute of Behavioral Neurosciences & Psychology, Fairfield, USA; 2 Internal Medicine, California Institute of Behavioral Neurosciences & Psychology, Fairfield, USA; 3 Neurological Surgery, California Institute of Behavioral Neurosciences & Psychology, Fairfield, USA; 4 Psychiatry, California Institute of Behavioral Neurosciences & Psychology, Fairfield, USA; 5 General Surgery, Shifa International Hospital, Islamabad, PAK; 6 Research Fellowship, Ventolini's Lab, Texas Tech University Health Sciences Center, Odessa, USA; 7 Research, Harvard Medical School, Boston, USA; 8 Otolaryngology - Head and Neck Surgery, California Institute of Behavioral Neurosciences & Psychology, Fairfield, USA; 9 Surgical Oncology, Tata Medical Centre, Kolkata, IND

**Keywords:** covid 19, itching, hypersensitivity pruritis, healthcare workers, occupational contact dermatitis, eczema, hand eczema, hand dermatitis, dermatitis, contact dermatitis

## Abstract

Occupational contact dermatitis (OCD) is an eczematous local inflammatory skin irritation caused by repeated use of hand sanitizer and other chemical substances. Occupational irritant contact dermatitis (OICD) and occupational allergic contact dermatitis (OACD) are the two variants of CD that cannot be identified clinically. Hand dermatitis (HD) is typically assessed as a clinical consequence because it affects the hands most frequently at work as per epidemiological studies on OCD. The Preferred Reporting Items for Systematic Reviews and Meta-Analyses (PRISMA) 2020 standards were followed when conducting this umbrella review. We used the search terms "Occupational Contact Dermatitis AND COVID-19" to search for the most pertinent papers in full text on the databases PubMed/MedLine, ScienceDirect, and PubMed Central (PMC). Additionally, the reference section of the papers was used to find more articles.

A total of 11,646 results were found, and eight papers remained after applying the inclusion criteria (full-text papers, English language, studies published in the previous 10 years, involving humans, and only systematic reviews). After completing the title and abstract screening, we obtained five papers. Next, the full-text screening and AMSTAR quality check were completed, yielding the same five papers. After searching ScienceDirect, five papers that met the inclusion criteria were included, and six papers were selected from the references, yielding a total of 11 papers. The causes of occupational dermatitis from protective face masks are discussed in this review. We anticipate an increase in the incidence of occupational dermatitis linked to face mask use given that a large segment of healthcare workers (HCWs) wear protective face masks. To understand the prevalence and available therapies for mask-related occupational dermatitis, further well-designed research is required.

## Introduction and background

Contact dermatitis (CD) is one of the most common occupation-related diseases in developed countries. It is a local inflammatory skin irritation caused by repeated exposure to local chemical or physical factors [1,2]. CD is classified into two types: irritant contact dermatitis (ICD) and allergic contact dermatitis (ACD). It is usually hard to clinically distinguish between them, and hence the diagnosis is usually based on the exclusion of ACD by using an allergic patch test [2]. The prevalence of occupational contact dermatitis (OCD) is estimated to be between 6.7% and 10.6%, which amounts to around 5.5/1000 person-years and a lifetime prevalence of 15%. Several people have lost their jobs due to this condition. Some of the most common risky jobs for OCD include agricultural work, construction work, healthcare-related jobs, hairdressing, as well as mechanics and machinists [3,4].

OCD can have a significant impact on costs and productivity at work. Affected personnel may need extended leave from work, retraining, relocation, and/or adjustments to work procedures. Employees can incur recurring non-reimbursed financial costs for treatments and preventative measures, such as moisturizers and soap alternatives [5]. OCD may limit social and home activities, which could have negative psychological impacts. Disparities in data gathering make research involving direct comparisons difficult. OCD is widely considered to be underreported due to a lack of physician access, detection, or diagnosis [5]. 

During the coronavirus disease 2019 (COVID-19) pandemic, some effective preventive measures were introduced, which have shown great efficacy in limiting the pandemic and reducing the rate of infection. Some of these preventive methods included the use of personal protective equipment (PPE) including gloves, masks, N95 respirators, goggles, gowns, and face shields. Other methods included the use of hand sanitizers and disinfectants. On the other hand, prolonged use of PPE combined with frequent hand washing or disinfecting can often result in adverse skin reactions (ASRs) as one of the most common dermatoses, which can result in a change in the epidermal barrier, as well as itching, pain, and burning [6].

Research indicates that OCD is a serious issue among working people, especially among healthcare workers (HCWs). Of note, 65.3% of HCWs were self-diagnosed with skin diseases, and 25.8% were contact/atopic dermatitis. HCWs who perform specific duties must have intact skin on their hands and forearms since it helps lower the risk of illnesses related to healthcare. Additionally, a variety of tasks related to healthcare have the potential to cause OCD, some of which may be severe and difficult to treat. For the health and safety of both patients and personnel, it is crucial to take into account the skin and skincare of HCWs. Working with damp hands and contact with soaps and cleaning supplies are the two most frequent causes of OCD [7]. 

Wearing PPEs and masks and using hand sanitizing persistently and over a long period will increase the risk of developing eczema, pruritus, urticaria, erosion, scaling, desquamation, and exudative lesions either in the hand or face. This systematic review aims to discuss how preventative measures that emerged during the COVID-19 pandemic can lead to CD either in the hand or face, and examine the preventive measures and treatment for these cases.

## Review

Materials and methods

Search Strategy

This systematic review was conducted according to the Preferred Reporting Items for Systematic Reviews and Meta-Analyses (PRISMA) 2020 guidelines [8]. We searched PubMed/MedLine, PubMed Central (PMC), and ScienceDirect by using the search terms “Occupational Contact Dermatitis AND COVID-19” to identify the most relevant papers. A search based on papers' references was also done. Papers were thoroughly searched using appropriate keywords like dermatitis, occupational dermatitis, contact dermatitis, and healthcare worker. 

Inclusion and Exclusion Criteria 

The inclusion criteria were as follows: peer-reviewed articles, published in the last 10 years, containing free full text, and involving patients who developed CD during the COVID-19 pandemic. The included papers are all in the English language, and focus only on humans; literature reviews, systematic reviews, and meta-analysis studies were included. Articles focusing on other dermatological diseases or patients who develop CD without COVID-19 as the risk factor, editorials, clinical trials, RCTs, studies whose full-text articles were unavailable, and animal studies were excluded (Table 1).

**Table 1:** Inclusion and exclusion criteria CD: contact dermatitis; COVID-19: coronavirus disease 2019

Inclusion criteria	Exclusion criteria
Peer-reviewed papers	Not peer-reviewed
Published articles	Unpublished literature
Free full-text papers	Full-text unaivalable
Literature reviews or systematic reviews and meta-analysis	Other study designs, editorials, and commentaries
Occupational dermatitis during the COVID-19 pandemic	Topics not related to occupational dermatitis or CD not associated with COVID-19
English language	Studies in other languages
Human studies	Animal studies

Quality Appraisal 

After applying the inclusion and exclusion criteria, quality assessment was done on the remaining systematic reviews by using the Assessing the Methodological Quality of Systematic Reviews (AMSTAR) checklist [9; for narrative reviews, we used Scale for the Assessment of Narrative Review Articles (SANRA) guidelines [10]. Only those articles that fulfilled more than 70% of the criteria of AMSTAR and SANRA checklist parameters were included in this study (Tables 2-3).

**Table 2:** SANRA quality assessment for literature reviews *Indicates the criteria fulfilled by the paper SANRA: Scale for the Assessment of Narrative Review Articles

SANRA checklist	Chu et al. [4]	Tang et al. [6]	Abdali and Yu [11]
Justification of the article’s importance for the readership	*	*	*
Statement of concrete aims or formulation of questions	*	*	*
Description of the literature search		*	
Referencing	*	*	*
Scientific reasoning	*	*	*
Appropriate presentation of data	*	*	*
Total	5/6	6/6	5/6

**Table 3:** AMSTAR quality assessment for systematic reviews and meta-analysis *Indicates the criteria fulfilled by the paper AMSTAR: Assessing the Methodological Quality of Systematic Reviews; PICO: patient/population, intervention, comparison, and outcomes; RoB 2: Cochrane risk-of-bias tool for randomized trials

AMSTAR checklist	Larese Filon et al. [1]	Schütte et al. [2]	Jacobsen et al. [3]	Keegel et al. [5]	Papadatou et al. [7]	Yu et al. [12]	Keng et al. [13]	Saary et al. [14]
Did the research questions and inclusion criteria for the review include the components of PICO?	*	*	*	*	*	*	*	*
Did the report of the review contain an explicit statement that the review methods were established prior to the conduct of the review and did the report justify any significant deviations from the protocol?	*	*	*	*	*	*	*	*
Did the review authors explain their selection of the study designs for inclusion in the review?	*	*	*	*	*	*	*	*
Did the review authors use a comprehensive literature search strategy?	*	*	*	*	*	*	*	*
Did the review authors perform study selection in duplicate?	*	*	*	*	*	*	*	*
Did the review authors perform data extraction in duplicate?	*	*	*	*	*	*	*	*
Did the review authors provide a list of excluded studies and justify the exclusions?		*		*	*		*	*
Did the review authors describe the included studies in adequate detail?	*		*		*	*	*	
Did the review authors use a satisfactory technique for assessing the risk of bias RoB in individual studies that were included in the review?	*	*	*	*	*	*	*	*
Did the review authors report on the sources of funding for the studies included in the review?	*	*	*	*	*	*	*	*
If meta-analysis was performed, did the review authors use appropriate methods for statistical combination of results?	*	*	*	*	*	*	*	*
If meta-analysis was performed, did the review authors assess the potential impact of RoB in individual studies on the results of the meta-analysis or other evidence synthesis?	*	*	*	*	*	*	*	*
Did the review authors account for RoB in individual studies when interpreting/ discussing the results of the review?	*	*	*	*	*	*	*	*
Did the review authors provide a satisfactory explanation for, and discussion of, any heterogeneity observed in the results of the review?	*	*	*	*	*	*	*	*
If they performed quantitative synthesis did the review authors carry out an adequate investigation of publication bias (small study bias) and discuss its likely impact on the results of the review?		*	*	*		*	*	
Did the review authors report any potential sources of conflict of interest, including any funding they received for conducting the review?	*	*	*	*	*	*	*	*
Total	14	15	15	15	15	15	16	14

Results

Search Results

**Figure 1:**
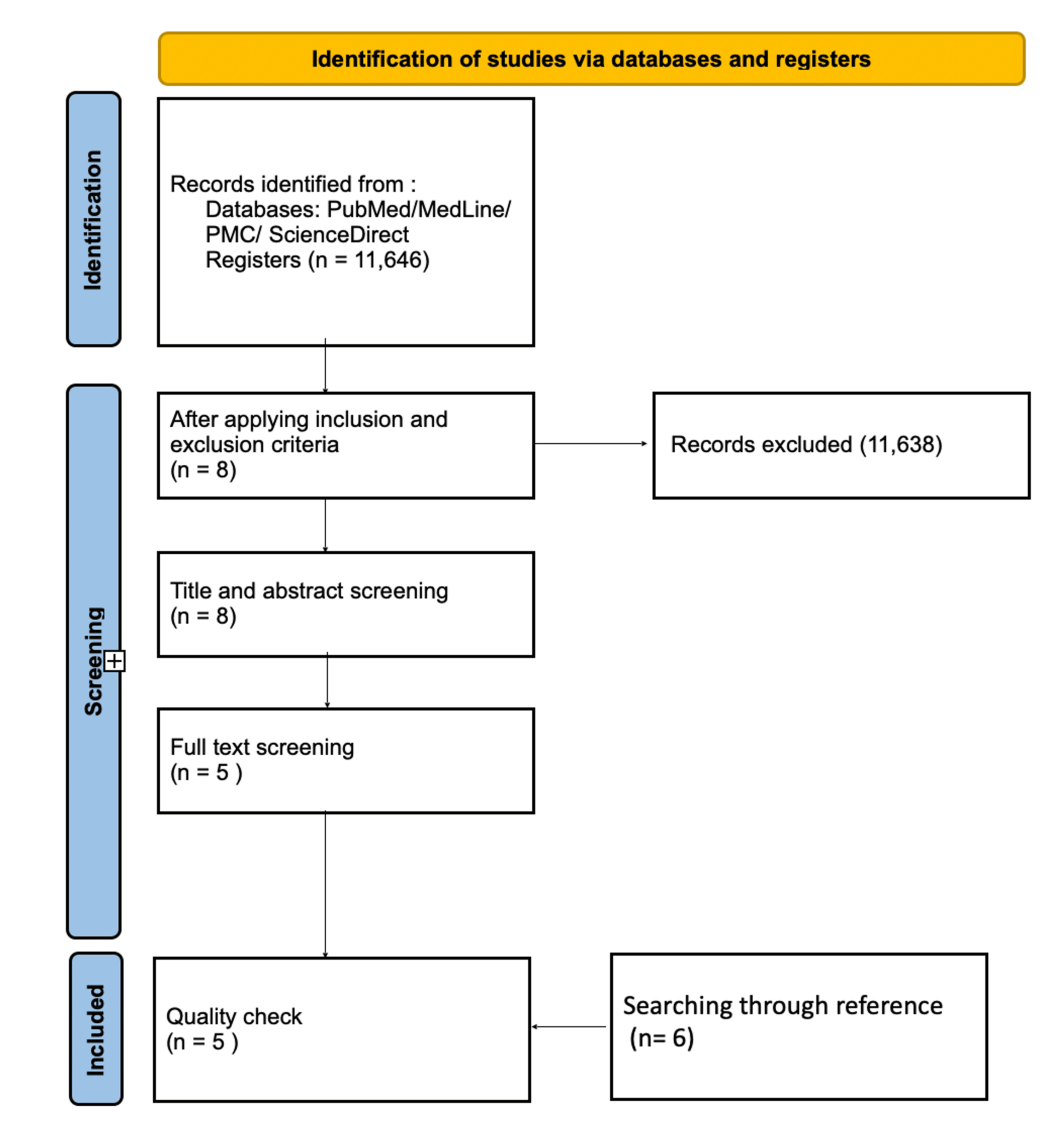
PRISMA flow chart depicting the selection of papers PMC: PubMed Central; PRISMA: Preferred Reporting Items for Systematic Reviews and Meta-Analyses

This review used the following databases: Pubmed, Medline, PMC, and ScienceDirect, and (11,646) results were identified. Eight papers remained after applying the inclusion criteria (full-text papers, English language, papers published in the last 10 years, involving humans, and only systematic reviews). Title and abstract screening was done and we ended up with five papers. After that, full-text screening and quality checks were based on AMSTAR for systematic reviews and SANRA for narrative reviews, and we were left with five papers. After searching in ScienceDirect, five papers were included that matched the inclusion criteria, and six papers were chosen from the references, giving us a total of 11 papers (Figure 1). 

Study Analysis

A summary of the details from the articles is presented below (Table 4).

**Table 4:** Summary of the included studies ACD: anemia of chronic disease; CD: contact dermatitis; CINAHL: The Cumulative Index of Nursing and Allied Health Literature; COVID-19: coronavirus disease 2019; EMBASE: Excerpta Medica Database; HCWs: healthcare workers; ICD: irritant contact dermatitis; OCD: occupational contact dermatitis; OIHD: occupational irritant hand dermatitis; OSD: occupational skin diseases; PPE: personal protective equipment; PRISMA: Preferred Reporting Items for Systematic Reviews and Meta-Analyses; RCT: randomized controlled trial; WSIB: Workplace Safety and Insurance Board

Author	Year	Title	Aim of the study	Methods	Results	Conclusions
Larese Filon et al. [1]	2021	Incidence of occupational contact dermatitis in healthcare workers: a systematic review	This study aimed to compile statistics on OCD incidence among healthcare workers	We used the search term "incidence of contact dermatitis in HCWs" per Preferred Reporting Items for Systematic Reviews and Meta-analyses (PRISMA) standards to search the databases PubMed/MEDLINE (1980–present), EMBASE (1980–present), and Cochrane Library (1992–present) through May 2020. With follow-up periods spanning 1987 and 2013, 16 studies (six cohorts; 10 register-based) met the inclusion criteria	According to research employing occupational disease records, the prevalence of OCD ranged from 0.6 to 6.7 per 10,000 person-years. According to the cohort studies, the incidence was higher in studies that included trainee nurses, ranging from 15.9 to 780.0 per 10,000 person-years. In contrast to other HCWs, a higher incidence was seen among dental professionals, particularly dental technicians and nurses	Our work emphasizes the need for future cohort studies in sizable occupationally exposed populations from various settings to properly estimate the burden of OCD in HCWs. Future research on this topic will be more accurate thanks to the OMEGA-NET consortium's promotion of occupational cohort harmonization. Due to the requirement that HCWs wear personal protective equipment for extended periods during the present COVID-19 pandemic—which raises the risk of OCD—the burden of OCD has assumed particular importance
Schütte et al. [2]	2023	Work-related and personal risk factors for occupational contact dermatitis: a systematic review of the literature with meta‐analysis	The goals of this systematic review were to (1) identify personal and workplace risk factors associated with CD risk estimations; and (2) group comparable personal and workplace risk factors for CD in a meta-analysis	This evaluation was carried out per a protocol that was already registered in PROSPERO. As personal characteristics and jobs were reviewed in addition to risk factors relating to the workplace, we departed from the procedure regarding the exposure	29 research with 846 209 people in total, 26 study populations, and 29 studies were found to be looking at 52 CD risk variables. Five risk factors, all of which were for irritating contact dermatitis (ICD), were the subject of meta-analyses. Wet work and ICD were shown to be associated with moderate-quality evidence	In conclusion, several personal and professional risk factors for CD were discovered. Our findings highlight the need to address both personal and workplace risk variables in efforts to prevent occupational CD
Jacobsen et al. [3]	2021	Causes of irritant contact dermatitis after occupational skin exposure: a systematic review	The aim is to conduct a thorough analysis and present: an evaluation of the risk of ICD concerning the kind, amount, severity, and length of exposure to occupational irritants (dose-response relation); an explanation of the disease's onset period concerning exposure and any potential threshold values (lower limit of effect); additionally, a prognosis for ICD is evaluated, along with how exposure continues to affect prognosis and how exposure stops having an influence	48 studies were included in the systematic review after 1516 titles were found through a systematic search	We discovered that there was high evidence for a connection between ICD and wet work, moderate evidence for detergents and non-alcoholic disinfectants, and strong evidence for a combination. The highest quality studies offered only weak support for a relationship between the usage of occlusive gloves and other wet work irritants in the absence of other exposures, and weak support in the presence of both	According to this research, there is significant support for a link between ICD and wet work exposure combined with non-alcoholic disinfectants, moderate support for metalworking fluid exposure, limited support for mechanical exposure, and strong support for a poor prognosis for ICD
Chu et al. [4]	2020	Occupational contact dermatitis: common occupational allergens	It is estimated that 6.7% to 10.6% of people have occupational contact dermatitis, which can result in missed work and job loss. While treatment might offer short-term respite, identifying the allergen causing the problem could assist the physician in advising on minimizing or avoiding exposure	-	-	Many professions carry a high risk of ACD, and there are even more potential offenders. Knowing the common allergens may help to guide the history and physical examination, as well as assist the astute clinician in selecting allergens for patch testing, even though obtaining the occupation is crucial as the first step in the evaluation of occupational ACD
Keegel et al. [5]	2009	The epidemiology of occupational contact dermatitis (1990-2007): a systematic review	Comparison of studies directly is made more difficult by differences in data collecting. There is a common assumption that there are a significant number of unreported OCD cases due to a lack of physician access, detection, or diagnosis. We give a thorough analysis of OSD research	Work-related skin disease, occupational dermatitis, and occupational eczema were searched for in the relevant literature from January 1980 to December 2007 using the Medline and Web of Science databases. The search was restricted to human-related materials in the English language. Additionally, we conducted a manual literature search in the Carlton, Victoria, Australia, libraries of the Skin and Cancer Foundation and the Occupational Dermatology Research and Education Centre		Different definitions, data collection techniques, and reporting systems, as well as the variation in the degree of industrialization, which results in variances in occupational exposures, are just a few of the factors for the national differences in the reported frequency of OCD. The incidence and prevalence of OCD are poorly covered in the literature, and the data that is currently accessible could not be accurate. We anticipate that as international CD interest organizations grow and span national and specialty boundaries, OCD diagnosis and reporting will become more standardized
Tang et al. [6]	2023	Contact dermatitis caused by prevention measures during the COVID-19 pandemic: a narrative review	Healthcare professionals spent a significant amount of time wearing masks, gloves, and goggles as part of their PPE during the COVID-19 outbreak. Public areas were sprayed with disinfectant to lessen the virus's routes of transmission. In addition, for hygienic reasons, the body, hands, and clothing were regularly cleaned and disinfected. According to studies, these habits run the risk of easily irritating the skin and weakening the skin barrier. CD can result from long-term irritation or allergy exposure	The National Library of Medicine (PubMed) and Web of Science databases were searched using the following subject headings: COVID-19; contact dermatitis; adverse skin reaction; protective personal equipment; dermatitis; mask; glory; hand hygiene, disinfection; face shield; goggle; protect cloth. The two databases yielded a total of 246 and 646 articles, respectively. Duplicate articles were removed, leaving 402 articles	This article examines cases of CD during the COVID-19 pandemic that were brought on by different hygiene and preventive measures. Better skin management education could reduce the amount of skin damage caused by COVID-19 prevention measures	PPE has turned into a weapon in the fight against the virus, and using disinfectants and hand cleaners correctly can help cut down on the disease's spread. But it's important to pay attention to the skin damage that COVID-19 preventive measures cause. Numerous studies have documented the harm it causes to the skin, with CD being one of the prevalent conditions
Papadatou et al. [7]	2016	Effectiveness of interventions for preventing occupational irritant hand dermatitis: a quantitative systematic review protocol	This systematic review's objective is to identify RCT and other quantitative research design findings that can add to the body of knowledge and help assess the efficacy of interventions for OIHD prevention	Finding both published and unpublished studies is the goal of the search strategy. A three-step search strategy will be used. Following a first, focused search of MEDLINE and CINAHL, text words from the title and abstract, as well as index terms used to characterize the articles, will be analyzed. Then, a second search will be conducted across all included databases using all found keywords and index phrases. Third, all recognized reports and papers' reference lists will be checked for new studies. Only research that has been published in English will be taken into consideration for this review	All wet workers may experience long-term illness due to occupational skin conditions, including OIHD, which can also harm their careers. For instance, some OSDs brought on by particular substances might lead to chronic skin illness, a higher risk of allergic dermatitis, the emergence of inflammatory problems like urticaria, or even ulcerative and degenerative skin diseases. Additionally, the presence of OSDs may harm how patients are treated and how much it costs the health services. For (i) developing a framework for the early detection of skin issues and (ii) managing the exposure to substances that have the potential to cause harm, reliable and ongoing health surveillance for those at risk of developing skin reactions is crucial	This evaluation will take into account studies that evaluate the following primary preventative strategies for OIHD in wet workers at work and home (before and after work): use of moisturizers, such as those with high and low lipid contents. Barrier creams, such as those that may include silicone, lanolin oil, liquid paraffin lotion, or hydrocarbon. Gloves (made of cotton or rubber). Education (such as seminars and training sessions; delivered in person or online). Due to the variety of regimens, this evaluation will take into account any dosage/intensity of preventative intervention for any period, including complex interventions that include multiple of the aforementioned interventions
Abdali and Yu [11]	2021	Occupational dermatoses related to personal protective equipment used during the COVID-19 pandemic	The increased infection control practices that the public and healthcare professionals implemented in the wake of the COVID-19 pandemic have resulted in a marked rise in the prevalence of documented occupational dermatoses. The most frequently reported occupational dermatitis is irritant contact dermatitis, which is typically brought on by frequent hand washing and the use of face-protecting masks and respirators. Strategies for relieving pressure, moisturizing the skin sufficiently, and using gentle skin care are crucial preventative measures for occupational dermatoses associated with personal protective equipment	-	-	The authors of this review addressed the prevalent forms of occupational dermatoses that have been documented as a result of increased PPE use and improved hygiene practices used by HCWs and non-HCWs to stop the spread of COVID-19. They also talked about ways to prevent, diagnose, and treat occupational dermatoses linked to personal protective equipment. PPE-related dermatoses will probably continue to be more common until a vaccine and treatments are developed that work for everyone
Yu et al. [12]	2021	Occupational dermatitis to facial personal protective equipment in health care workers: a systematic review	The goal of this study was to provide a thorough assessment of occupational dermatoses in HCWs caused by N95 respirators and medical face masks	Using the PubMed and Embase databases, a systematic review was carried out per the PRISMA criteria. Articles were considered if they discussed occupational dermatoses brought on by N95 respirators, surgical/procedure masks, or both	16 of the 344 articles we found may be included in this review. A few articles concentrated on the facial occupational dermatoses of healthcare professionals. There have been reports of allergic contact dermatitis to glue, elastic straps, and formaldehyde emitted from the mask cloth. Due to pressure and friction, irritant contact dermatitis was frequently seen on the cheekbones and nasal bridge. Long-term mask use (>6 hours) and a personal history of atopic dermatitis were linked to irritable dermatitis	The American Contact Dermatitis Society members who conducted this systematic analysis emphasize instances of occupational dermatitis to face protection equipment, including possible offending allergens. This research may aid in the diagnosis and care of healthcare professionals who suffer from facial occupational dermatitis
Keng et al. [13]	2021	Personal protective equipment-related occupational dermatoses during COVID-19 among health care workers: a worldwide systematic review	We conducted a thorough, systematic evaluation of the effects and burden of PPE-related dermatoses on HCWs around the world as part of our study to fill this gap in the literature. As PPE usage grew, we recorded the occurrences of various skin lesions and suggested remedies to reduce the negative skin reactions experienced by our HCWs throughout this ongoing epidemic	For articles on PPE-related dermatoses in healthcare workers during the COVID-19 pandemic that were authored in English and published between January 1, 2020, and January 30, 2021, online databases were examined	A total of 3958 participants from 16 trials were included. The most prevalent dermatoses, which mostly affect the hands and face, are xerosis, pressure-related erythema, and contact dermatitis. The contactants that were most frequently implicated were gloves, N95 masks, goggles, and enhanced hand hygiene frequency	The difficulties created by PPE-related occupational dermatoses can be greatly decreased by actions such as regular basic skin care education, early access to specialty clinics via telemedicine, and the development of better-fit PPE
Saary et al. [14]	2005	A systematic review of contact dermatitis treatment and prevention	A program of care for OCD has been created by Ontario, Canada's WSIB. This necessitated the use of evidence-based treatment choices. Given the importance of OCD and the historical inability to alter the clinical result, WSIB requested an independent evaluation of the literature to assist in the development of recommendations based on the best available research	Systematic searches were performed on numerous databases. Articles were given an excellent, fair, or bad rating based on established quality evaluation criteria and independent double review. Data on treatment benefits were collated, and conclusions were drawn based on how strongly the available evidence was assessed	49 studies overall met the criteria for inclusion. CD caused by irritants is prevented with barrier lotions with dimethicone or perfluoropolyether, cotton liners, and softened materials. Moisturizers high in lipids both treat and prevent irritating CD. Rhus dermatitis is prevented with quaternium 18 bentonite (organoclay) and topical skin protection. Chelator cream, which contains diethylenetriamine penta-acetic acid, guards against nickel, chromium, and copper dermatitis	There are a few therapies that can effectively cure or prevent irritant and allergic CD, but more well-controlled, outcome-blinded research is required, especially in the field of allergic CD prevention

Discussion

This study involved a systematic review to analyze 11 papers related to OCD due to COVID-19 infection, an assessment of the risk factors of COVID-19 and its PPE in the development of OCD in the hand and face, the COVID-19 risk factors for developing OCD, besides its incidence, preventative measures and treatments, and effect of OCD on healthcare personnel assessment of personal protective equipment PPE related to OCD during COVID-19. Keegel et al. have described the incidence and prevalence of OCD in Western countries like Germany, Sweden, the USA, Denmark, Finland, Australia, the UK, and also Singapore. The prevalence of CD is high (reaching 90-95%) and is most commonly associated with OSD [5]. In Germany, Finland, and Denmark, reporting is required. In Saarland and Northern Bavaria, Germany, standardized records for OSD have been established. Based on them, there were seven instances of OSD per 10000 workers per year, a close approximation for the entire working population of Germany. As time went on, the incidence declined from 10.7 per 10000 workers in 1990-92 to 4.9 per 10000 workers in 1993-99 [5]. 

In Finland, physicians are required to document all instances to a central registry; between 1990 and 1993, 4365 cases of ICD/ACD or CU were identified from reported cases [5]. In the eight years between 1984 and 1991, the Danish Register of Occupational Diseases estimated a prevalence of 17700 instances of OSD among 2.6 million Danish workers. Only 11% of recognized OSD cases received compensation during this time, with an overall incidence of eight instances per 10,000 workers per year [5]. In a 1983 Swedish study, 16584 people (7.6% of the population of working age) responded to a questionnaire via mail (response rate: 82.9%); 1238 of these people had hand dermatitis (7.5%); 13.0% of part-time workers and 10.3% of full-time workers reported having hand dermatitis [7].

While Germany only includes cases that have been reviewed by occupational government physicians, Finland and Denmark report all cases [5]. In the USA, 30074 people took part in the National Health Interview Survey, which also included in-person interviews with members of randomly chosen families. The prevalence estimate for one year was 170 instances per 10000 workers. Additionally, information produced by employers was used to collect data. The US Bureau of Labor Statistics conducts yearly polls of almost 250000 carefully chosen companies. Up to 23% of workers held jobs beyond the scope of this survey. Annual incidence in 1991 was 7.7 cases per 10000 workers (range: 8.1 to 6.7 cases per 10000 from 1993 to 1997). The number of actual cases may be significantly higher due to shortcomings in recognition and reporting [5]. In contrast, there are differences in the incidence rates for voluntary systems. In comparison to the UK (1.3 per 10000) and Australia (2.2 per 10000), the Netherlands reported significantly higher rates (15 per 10000). The most accurate method for measuring the incidence of workers' compensation avoidance may be voluntary reporting programs. In the UK, occupational physicians and dermatologists were employed as correspondents [5]. 

Contact Dermatitis Caused by Personal Protective Equipment During the COVID-19 Pandemic

Facial masks including surgical masks, cloth masks, and N95/KN95 respirators cause ASRs, and eventually CD; its ear bands can cause ACD. However, cloth masks can cause fewer ASRs in contrast to others. As per Tang et al. [6], prolonged wearing of masks causes skin contact, which can cause various types of skin ASRs, such as redness, pruritus, and acne; 6.2% of HCWs were diagnosed with CD. The most commonly affected areas were the nose bridge, ears, cheeks, perioral area, and chin [6]. People who wore gloves for more than eight hours have complained of skin irritation. It is better not to increase the number of layers of latex gloves, to reduce skin damage [6].

During the COVID-19 pandemic, 67% of HCWs used goggles for more than four hours. The most common side effects resulting from using goggles are physical stress-related skin lesions, mechanical friction, and finally CD. Skin lesions may progress to erythema, and depression to erosion and ulceration but the most common skin lesions are pressure sores and rashes [6]. Face shields account for 23% of all skin problems that are caused by PPE, and the forehead is the most affected site. They can cause itching, pain, abrasion, and changes in skin properties [6]. While protective clothing is rarely considered to cause CD (3.6%), dry skin and pruritus are the most common complaints due to prolonged friction and pressure irritation [6].

Contact Dermatitis Caused by Hand Hygiene Products During the COVID-19 Pandemic

During the COVID-19 pandemic, frequent hand washing with soap or hand sanitizer and alcohol-based hand disinfection has led to the development of ICD and ACD. Hand hygiene products contain a variety of chemical additives, such as fragrances and disinfectants, which may be the cause of the rise in hand ACD. Propanol, ethanol, quaternary ammonium salts, iodine, chlorhexidine, triclosan, sodium benzoate, phenoxyethanol, and sterols are common allergens or irritants. Proteins become denatured as a result of isopropanol's disruption of the lipid bilayer structure between cells. Dermatitis may be exacerbated by triclosan, chlorhexidine, and quaternary ammonium compounds, according to some studies [6].

The risk of eczema and dry hands is increased by hand washing with hand hygiene products for longer than 10 seconds, more than eight times a day. Maintaining good hand hygiene and disinfecting hands can also cause new skin problems and worsen pre-existing eczema. During the COVID-19 pandemic, the majority of healthcare workers washed their hands more than 10 times a day (20 seconds on average). Public servants who are required to practice good hand hygiene are also susceptible to hand skin damage [6].

Contact Dermatitis Due to Non-COVID-19 Risk Factors

Personal risk factors like wet work demonstrated some weak evidence of an elevated risk for ICD. Based on low-quality cohort research, risk factors such as aliphatic amines, isocyanates, acrylates, and persulfate showed a substantial increase in CD risk when analyzing single risk estimate(s). Within the same study, eight work-related factors - herbicides, insecticides, fungicides, exhaust fumes, latex, textile dust, molds/fungi, and endotoxins - showed a substantial decrease in CD risk [5]. Disinfectants, wood dust, pharmaceutical medications, animals, food, plants, mites and insects, enzymes, and dry skin soiling are nine of the 28 work-related risk variables that were discovered, although none of them were significantly associated with an increased risk for CD [5].

Overall, the evidence for a causal relationship between wearing occlusive gloves without being exposed to other irritants and developing an ICD was rated as limited; the evidence for a causal relationship between wearing occlusive gloves and being exposed to other irritants was rated as moderate [2]. The overall evidence for a causative link between mechanical exposures and ICD was rated as limited, whereas the evidence for a causal association between MWF exposures and ICD was rated as considerable [2]. However, the occupational risk factor estimates for 24 occupational groups were taken from the papers that were included. Healthcare workers, hairdressers, blue-collar employees, painters, metal workers, and cleaners all had a considerably elevated risk of CD in two or more trials (a range of risk estimations demonstrated a significantly increased effect) [5].

The Effect of COVID-19 and Personal Protective Equipment on Patients With Contact Dermatitis

According to the studies by Yu et al. [12] and Keng et al. [13], the effect of OCD in HCWs from face masks was related to four common problems, and they suggest that reactions from facial PPE are mostly due to ICD. Adverse cutaneous reactions like medical face mask-related cutaneous responses have not been extensively studied. Although precise diagnoses were not given, we found three papers that focused on facial PPE usage during coronavirus infections. Although it is uncommon for dermatology journals to use the terms "adverse cutaneous reaction" and "skin damage," it is possible that non-dermatologists were surveyed in the absence of a clear diagnosis, perhaps only in populations affected by an epidemic or pandemic. The cheekbones, nasal bridge, and forehead were mask-related sites of engagement in this research, and they could be possible areas of attention for preventative workplace interventions. HCWs with a higher risk of negative reactions during COVID-19 wore PPE for more than six hours each day [12]. 

Allergic contact dermatitis can be caused due to rubber accelerators, which are found in mask elastic bands and are used to quicken the vulcanization of rubber; these have been linked to allergies. N-isopropyl-N'-phenyl para-phenylenediamine, one of the rubber antioxidants introduced during the vulcanization process, has been linked to mask-associated ACD. Masks are molded to the face using metal wires or rims. Adhesive chemicals are utilized in the manufacture of medical face masks and N95 respirators. Nickel ACD has been characterized as mask-associated ACD, and nickel and cobalt have also been identified as potential sources of ACD in protective equipment [12].

Irritant contact dermatitis, considered to be the most prevalent type of occupational skin disease, is caused by cytotoxic damage from close contact with irritants or chemicals. ICD symptoms include erythema, scaling, edema, and vesicles, as well as ulcerations and fissures at the site of contact. The severity of the condition is based on the irritant and duration of the exposure. In HCWs, occupationally linked ICD is most frequently recorded on hands. The cheeks and nasal bridge are frequently included in reports of cutaneous reactions during pandemics, which are mostly due to exposure to face masks. Prolonged usage of the mask is listed as an additional risk factor in the studies in our review [12]. Meanwhile, acneiform eruption and contact urticaria are caused by of long-term use of face masks by HCWs, which has been linked to acne, most likely as a result of rubbing (acne mechanica) or occlusion. In one case series, acne-prone patients had a history of the condition. Contact urticaria is infrequently recorded, and the literature lacks case specifics [12].

Facial dermatitis is one of the most common skin injuries associated with PPE. Acne and pressure-related skin lesions are two examples of facial dermatoses linked to PPE. Going without goggles or a N95 mask for an extended period can result in pressure-related skin damage. Erythema and skin indentation are possible early symptoms. The affected areas may develop into cracks, erosions, blisters, or ulcers if appropriate protective measures are not implemented. The bridge of the nose and the cheeks are areas that are particularly vulnerable to pressure. In addition, abrasions and skin maceration at these locations may weaken the barrier of protection and cause a secondary infection [13].

Mask-related acne is another commonly observed cutaneous symptom. Due to increased heat and humidity, wearing masks and goggles frequently results in an excessive buildup of sweat and oil on the face. In regions with tropical climates, this effect can be even more pronounced. Furthermore, wearing a mask repeatedly might cause friction and pressure that can cause mechanical damage as well as sebaceous duct occlusion and microcomedone rupture. Overall, this may aggravate the already existing acne vulgaris and cause mechanical acneiform eruptions in those who have never had them [13]. 

Prevention

Several barrier lotions, moisturizing creams, and the usage of softened materials are useful in avoiding the onset of ICD as per high-quality trials. Short-term usage of specific moisturizers and barrier creams, as well as the use of cotton glove liners, were all found to be helpful in studies of fair quality. Interventions in education showed less promise [14]. According to Saary et al. [14], there are more than 37 different preventative methods including corticosteroids, nonsteroidal medications such as macrolide immunomodulators, barrier creams, emollients, natural or herbal products, glover-related interventions, modifications to work process or environment, and psychosocial or educational interventions. They suggest that moisturizing creams are effective in preventing the development of CD. Their study examines the ability of pretreatment with various concentrations of perfluoropolyether phosphate (Fomblin HC/P2) in various gel bases to prevent experimentally induced ICD due to four different irritants. Studies found 5% HC/P2 to be effective in preventing ICD. On the other hand, one study showed that a barrier cream with aluminum chlorohydrate as its active component was unsuccessful in preventing ICD and, in fact, worse than a vehicle control on capacitance tests. 

The role of moisturizers in the prevention of ICD was studied in two high-quality studies [14]. High- and low-lipid-content moisturizers are effective in preventing CD, especially the high-lipid moisturizer, which shows a major effect on CD prevention as measured by transepidermal water loss (TEWL), capacitance, chromameter, and clinical scores, compared with a lower-lipid content moisturizer that only showed a preventive effect with capacitance measures [14]. Fabric softeners were generally effective as per four trials of mediocre quality. A high-quality study examined whether fabrics treated with fabric softener caused experimentally inflamed and normal but sensitive skin to become less irritated compared to untreated fabrics. The intervention consisted of two repetitions of rubbing a damp towel on the forearm (either untreated or treated with fabric softener), followed by three parts of air drying per day for five days. The results showed that in comparison to untreated fabrics, skin exposed to fabrics treated with fabric softener had improved stratum corneum structure, barrier function, and hydration. Although the study's methodology might mimic towel use, it might not be a good representation of how people dress [14]. 

In our review, none of the studies looking at educational requirements reached good-quality standards. The efficacy of educational interventions in avoiding ICD was investigated in two fair-quality cohort studies. Participants in the intervention group among nursing home employees outperformed the control group on a quiz and demonstrated a stronger behavioral change in three of the six areas that were specifically targeted for improvement. Although the clinical examination was not blinded and was therefore potentially biased given the divergent results between the assessors and a blinded dermatologist, self-reported symptoms did not differ substantially between the groups. In a different study, which also looked into an educational intervention among auxiliary nurses, neither clinical nor bioengineering. TEWL assessments revealed a significant difference between the intervention and control groups [14]. 

Papadatou et al. [6] have suggested other preventative measures, like vocational rehabilitation, which refers to anything that helps an employee with a health condition or disability to return, stay in, or begin work. There is much evidence to suggest that employment is beneficial to one's health and that the advantages of working outweigh the risks associated with employment and the consequences of unemployment and joblessness. Promoting health while maintaining a work-life balance is essential to keeping employees healthy at work [6]. Papadatou et al. [6] also suggest other preventative measures to be taken into consideration, such as rehabilitation intervention, safety regulations, compliance with health, and incorporating biophysical factors to support employees in return to work or stay healthy while working [6].

Treatment

According to Saary et al. [14], lipid-rich moisturizers work well for the temporary relief of experimentally induced ICD. A moisturizing cream with 5% urea and 5% hydrogenated canola oil was applied twice daily for 14 days to an artificially generated ICD site. Comparing a treated site to an untreated control site, there was a significant improvement in barrier function (TEWL), skin hydration, and clinical assessments [14]. Researchers looked at six different types of moisturizers and discovered that they are generally successful for both clinical and bioengineering measures. Lipid-rich moisturizers like petrolatum showed better efficacy than less lipid-rich moisturizers. Evidence has shown that lipid-rich moisturizers work well in the immediate term. Another study of fair quality looked at the effects of moisturizing dry hands before gloving. While visual inspection revealed less dryness, neither TEWL nor erythema showed a change from control. Quality of life or return to work were not utilized as outcome measures in any of the studies [14]. 

Limitations

This study has a few limitations. Since only a few studies were included (11), caution should be exercised in interpreting our findings related to the causes and prevention of occupational dermatitis. Only a limited number of systematic reviews were available, leading to limitations in the findings reached. In addition, the studies included in this article have several limitations, such as some papers using only two search databases, and a few only involving one retrospective cohort research and a few cross-sectional surveys. Finally, some papers deviated from the protocol because it was not possible to recruit information specialists for planning and peer reviewing.

## Conclusions

In this review, we analyzed the causes of occupational dermatitis due to the use of protective face masks. Given the high number of HCWs who wear protective face masks, we predict an increase in the incidence of occupational dermatitis associated with face mask use. Further well-designed research is necessary to comprehend the prevalence and available treatments for mask-related occupational dermatitis. This review offers compelling evidence regarding the connections between irritating exposures and the onset of OICD related to wet job exposure, with or without the accompanying exposure to detergent and disinfectants. Regarding mechanical exposures and gloves, the evidence for metalwork exposures was moderate and sparse. Additionally, this analysis offers strong evidence for a poor prognosis of complete healing under unchanged exposure conditions and moderate evidence for a complete recovery under cessation or reduction of exposure. Several personal and professional risk factors for CD were discovered. Our findings highlight the need to address both personal and workplace risk variables in the efforts to prevent OCD.
